# Effect of different surgical approaches on the survival and safety of Siewert type II esophagogastric junction adenocarcinoma: a systematic review and meta-analysis

**DOI:** 10.1186/s12885-023-11640-5

**Published:** 2023-11-21

**Authors:** Hongyang Zheng, Xingmei Yin, Tiewen Pan, Xiandong Tao, Xiaolin Xu, Zhenjia Li

**Affiliations:** 1 Department of Thoracic Surgery, Third Affiliated Hospital of Naval Medical University, Shanghai, 201805 China; 2 Department of Blood Transfusion, Third Affiliated Hospital of Naval Medical University, Shanghai, 201805 China; 3 Department of Digestive Surgery, Shanghai Songjiang District Central Hospital, No. 746 Zhongshan Middle Road, Songjiang District Shanghai, Shanghai, 201600 China

**Keywords:** Transthoracic, Transabdominal, Siewert type II, Esophagogastric junction adenocarcinoma, Meta-analysis

## Abstract

**Background:**

Whether a transthoracic (TT) procedure by a thoracic surgeon or a transabdominal (TA) by a gastrointestinal surgeon is best for Siewert type II esophagogastric junction adenocarcinoma (EGJA) remains unknown. Survival and perioperative outcomes were compared between the two groups in this meta-analysis to clarify this argument.

**Methods:**

We searched 7 databases for eligible studies comparing TT and TA procedures for Siewert type II EGJA. The final analyzed endpoints included intraoperative and hospitalization outcomes, recurrence, complication, and survival.

**Results:**

Seventeen studies involving 10,756 patients met the inclusion criteria. The TA group had higher rates of overall survival (OS) (HR: 1.31 [1.20 ~ 1.44], *p* < 0.00001) and disease-free survival (DFS) (HR: 1.49 [1.24 ~ 1.79], *p* < 0.0001). The survival advantage of OSR and DFSR increased with time. Subgroup analysis of OS and DFS suggested that TA remained the preferred approach among all subgroups. More total/positive lymph nodes were retrieved, and fewer lymph node recurrences were found in the TA group. The analysis of perioperative outcomes revealed that the TA procedure was longer, had more intraoperative blood loss, and prolonged hospital stay. Similar R0 resection rates, as well as total recurrence, local recurrence, liver recurrence, peritoneal recurrence, lung recurrence, anastomosis recurrence and multiple recurrence rates, were found between the two groups. The safety analysis showed that the TT procedure led to more total complications, anastomotic leakages, cases of pneumonia, and cases of pleural effusion.

**Conclusions:**

The TA procedure appeared to be a suitable choice for patients with Siewert type II EGJA because of its association with longer survival, fewer recurrences, and better safety.

**Supplementary Information:**

The online version contains supplementary material available at 10.1186/s12885-023-11640-5.

## Introduction

In Western countries, the incidence of esophagogastric junction adenocarcinoma (EGJA) has increased significantly each year [1, 2]. Compared with esophageal and gastric cancer, its therapeutic effect is unsatisfactory, and one of the important reasons is that the treatment methods are not uniform or standardized, especially the surgical methods [3]. The classification system reported by Siewert et al. has been widely accepted in clinical practice in the past 20 years [4]. Esophagectomy + proximal gastrectomy (transthoracic [TT] or thoracoabdominal) is suitable for Siewert type I EGJA, and extended gastrectomy + distal esophagus resection (transabdominal [TA]) is suitable for type III EGJA [5]. However, for Siewert type II EGJA, whether esophagectomy + proximal gastrectomy performed in the TT procedure is better than extended gastrectomy + distal esophagus resection performed in the TA procedure has been debated by thoracic surgeons and gastrointestinal surgeons for decades.

In clinical studies, there were also notable differences regarding this argument. Chen et al. reported that the TA approach was associated with a longer overall survival (OS) time than the TT approach [6]. Voron et al. reported longer disease-free survival (DFS) in the TA group [7]. The survival advantages of the TA group were also found in some other studies [8, 9]. Longer survival may be associated with better lymph node dissection and fewer complications (anastomotic leakage, pneumonia, etc.) [9–11]. However, Blank et al. reported an opposite survival result [12]. In some other studies, no survival differences were found between the two groups [13, 14].

To clarify this clinical debate, the survival rate, recurrence rate, and perioperative outcomes were compared between the two groups in this meta-analysis.

## Materials and methods

Throughout the implementation of this study, the Preferred Reporting Items for Systematic Review and Meta-Analysis (PRISMA) statement was used as a checklist. (Table S1). (This study has been registered in PROSPERO, ID: CRD42023401527)**.**

### Search strategy

PubMed, ScienceDirect, The Cochrane Library, Scopus, Ovid MEDLINE, EMBASE, Web of Science, and Google Scholar were searched to find relevant literature published from their inception to January 2023. We used text and medical subject headings (MeSH) terms as follows: “Transthoracic”, “Transabdominal”, and “esophagogastric junction adenocarcinoma” (details are listed in Table S2). We also hand-searched the references of the included studies for further relevant articles.

#### Selection criteria

Inclusion criteria:

1. Population: Patients with Siewert type II EGJA.
2. Intervention and comparison: TT (Surgery procedure of digestive tract: esophagectomy, proximal gastrectomy and esophagogastrostomy; Range of lymph node dissection [LND]: two-fields lymphadenectomy. Different transthoracic approaches [Left single incision or thoracoabdominal two incisions], managements of residual stomach [gastric tube or not] and surgical forms [traditional open surgery or minimally invasive surgery] are all acceptable) vs. TA (Surgery procedure of digestive tract: extended gastrectomy with distal esophagus resection, and esophagojejunostomy; Range of LND: D1+ or D2 lymphadenectomy. Different surgical forms [traditional open surgery or minimally invasive surgery] are all acceptable).
3. Outcomes: Intraoperative and hospitalization outcomes, recurrence, complication, and survival.
4. Study design: Cohort study (CT) or RCT.

Exclusion criteria: basic/animal-based study, review, meta-analysis, abstract only, and study lacking the data of the above outcomes.

### Data extraction

The following data were extracted by two independent investigators (HYZ and YMY): participant characteristics, intraoperative and hospitalization outcomes (operating time, intraoperative blood loss, etc.), recurrence (total, local, lymph node recurrence, etc.), complications (total complication, complication [Clavien-Dindo classification III–IV], postoperative mortality, etc.) [15], and survival (OS, DFS, etc.). Disagreements were resolved by the above two investigators through recheck and discussion.

#### Outcome assessments

As a supplement to survival data (OS, PFS), we also analyzed the survival rate at 1, 2, 3, and 5 years. Subgroup analysis of OS and DFS was performed according to published year, region, TT group, surgical volume, and study design.

### Quality assessment of the included studies

The Jadad Scale (5 points) was used to assess the RCTs. The assessment tool focused on three main items: accountability of patients, randomization, and masking. Studies of high quality scored three or more [16].

The Newcastle–Ottawa Scale (NOS, 9 points) was used to assess the CTs. The assessment tool focused on the following criteria: selection (four points), comparability (two points), and exposure (three points). Studies of high quality scored eight or nine points, and studies of medium quality scored six or seven points [17].

The evidence level of the results was assessed by the Grades of Recommendations Assessment, Development and Evaluation (GRADE) system based on publication bias, inconsistency, indirectness, risk of bias, and imprecision [18].

### Statistical analysis

All statistical analyses were performed using STATA 12.0 software and Review Manager 5.3. The pooled risk ratio (RR) was used to analyze dichotomous variables (recurrences, complications, etc.). The mean difference (MD) was used to analyze continuous variables (intraoperative blood loss, operating time, etc.). The hazard ratio (HR) was used to analyze survival data (OS and DFS). In the analysis of advantageous outcomes (OS, number of lymph nodes retrieved, etc.), RR > 1, MD > 0, or HR < 1 suggested that it was beneficial to the TT group. In the analysis of disadvantageous outcomes (recurrences, intraoperative blood loss, etc.), RR > 1 or MD > 0 suggested that it was beneficial to the TA group. *I*^*2*^ and Cochran’s Q test were used to assess interstudy heterogeneity. When the *p* value was > 0.1 and the *I*^*2*^ value was ≤ 50%, a fixed-effects model was used; otherwise, a random-effects model was applied. Funnel plots were conducted to assess publication bias. A *p* value < 0.05 indicated that the results were significantly different.

## Results

### Study identification and characteristics

**Fig. 1:**
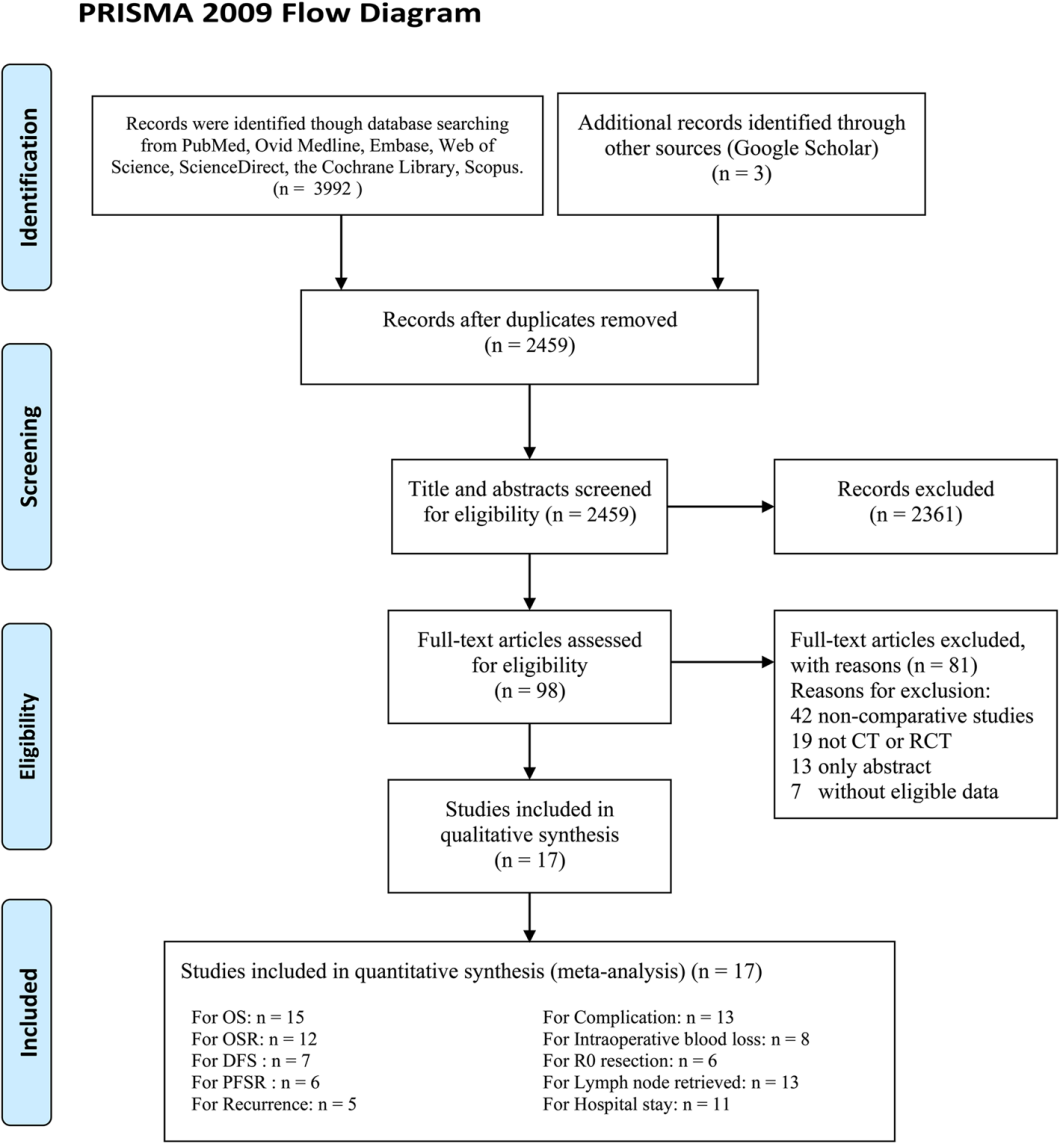
Flow chart of the study selection process

After screening 2459 relevant publications, we included 17 studies for the meta-analysis, of which 15 studies were CTs and the other 2 studies were RCTs (Fig. 1) [6–14, 19–26]. These 17 studies included 10,756 patients in total, including 8026 in the TA group and 2730 in the TT group. Table 1 summarizes the characteristics of the 17 studies. Table S3 summarizes the quality assessments of the included studies, in which 9 studies were of high quality and 8 studies were of medium quality. Table 2 summarizes the evidence level assessments of the results, in which all evidence levels were very low or low.

**Table 1:** Summary of the baseline characteristics of the included studies

Study		Country	Period (year)	Groups	Patients	Sex (M/F)	Age (Mean or median, year)	Tumor size (cm)	Pathological TNM stage				Outcomes^a^	Study design
									I	II	III	IV		
2022	Oh [10]	South Korea	2001.01-2019.05	TT group	46	37/9	61	3.5	15	17	13	1	①②③④⑥⑦⑧⑨⑩⑪⑫	CT
				TA group	126	96/30	61	4.0	48	41	35	2		
2022	Chen [6]^b^	China	1973–2020	TT group1	5657	4439/1218	-	-	274	2448	2813	122	①⑫	CT
				TT group2	942	762/180	-	-	42	397	454	49		
				TA group	504	399/105	-	-	42	146	274	42		
2020	Xing [8]	China	2009.11-2018.03	TT group	30	26/4	61.8	5.3	20	0	10	0	①②③④⑤⑥⑦⑧⑪⑫	CT
				TA group	181	151/30	61.9	4.1	18	101	62	0		
2019	Voron [7]	French	1997.01-2010.03	TT group	119	105/14	61.3	-	33	24	62	0	①②③④⑤⑧⑨⑩⑫	CT
				TA group	64	58/6	63.5	-	28	8	28	0		
2019	Tosolini [13]	Germany	2000–2013	TT group	91	82/9	58.8	-	3	20	62	6	①②③④⑤⑧⑫	CT
				TA group	179	146/33	63.6	-	4	55	115	5		
2019	Reddavid [14]	Italy	2000.01-2017.01	TT group	140	124/16	65.5	-	13	30	87	10	①③⑧⑨⑪⑫	CT
				TA group	60	53/7	66.8	-	9	23	17	11		
2018	Yang [11]	China	2004–2014	TT group	81	60/21	64	4.2	12	34	35	0	①③⑥⑦⑧⑨⑪⑫	CT
				TA group	77	55/22	62	4.7	13	31	33	0		
2018	Blank [12]	Germany	2001–2015	TT group	56	48/8	66.5	-	-	-	-	-	①⑤⑧⑨⑩⑪⑫	CT
				TA group	186	138/48	64.7	-	-	-	-	-		
2016	Zhang [9]	China	2006.01-2009.12	TT group	109	92/17	63.4	-	8	31	70	0	①③⑥⑦⑨⑩⑫	CT
				TA group	199	160/39	62.5	-	23	42	134	0		
2015	Zhou [19]	China	2007.07-2012.07	TT group	140	119/21	65	5.5	3	95	42	0	①③⑥⑦⑧⑨⑪⑫	CT
				TA group	194	163/31	67	5.7	7	131	56	0		
2015	Kurokawa [20]	Japen	1995.07-2003.12	TT group	85	63/22	63	7.0	-	-	-	-	①②③④⑤⑥⑦⑨⑩⑫	RCT
				TA group	82	71/11	60	6.2	-	-	-	-		
2012	Reeh [21]	Germany	1992–2009	TT group	51	48/3	62	-	6	12	27	6	①②③④⑤⑧⑨⑪	CT
				TA group	38	33/5	63	-	4	9	17	8		
2012	Ovrebo [22]	Norway	1984–2000	TT group	33	26/7	61	-	-	-	-	-	①⑪⑫	CT
				TA group	55	49/6	70	-	-	-	-	-		
2010	Zheng [23]	China	1994.01-2003.12	TT group	284	204/80	60.7	-	2	107	116	59	①③⑥⑦⑧⑪⑫	CT
				TA group	47	31/16	56.4	-	0	12	23	12		
2007	Omloo [24]	Netherlands	1994.04-2000.02	TT group	110	95/15	62	-	-	-	-	-	①②③⑤⑩	RCT
				TA group	95	83/12	65	-	-	-	-	-		
1999	Wayman [25]	UK	-	TT group	20	17/3	63	-	-	-	-	-	⑥⑦⑧⑨⑪⑫	CT
				TA group	20	17/3	71	-	-	-	-	-		
1998	Graham [26]	UK	1985–1989	TT group	32	-	-	-	-	-	-	-	⑥⑫	CT
				TA group	119	-	-	-	-	-	-	-		

*CT *Cohort study, *M/F *Male/female, *RCT *Randomized clinical trial, *TA *Transabdominal, *TNM *Tumor Node Metastasis, *TT *Transthoracic, *UK *United Kingdom

^a^ Outcomes: ① Overall survival; ② Disease-free survival; ③ Overall survival rate; ④ Disease-free survival rate; ⑤ Recurrence; ⑥ Operating time; ⑦Intraoperative blood loss; ⑧ Number of lymph node retrieved; ⑨ Number of positive lymph node retrieved; ⑩ R0 resection; ⑪ Hospital stay; ⑫ Complication

^b^ In study Chen 2022[6], TT group was divided into two groups:1. TT group1: Left intercostal thoracotomy approach; 2. TT group2: Right intercostal thoracotomy approach + median laparotomy

**Table 2:** GRADE quality assessment by therapeutic strategy and study design for the outcomes

Primary outcomes	No. of Studies	No. of Participants		Differences(95%CI)^a^	Quality Assessment					Quality
		TT group	TA group		Risk of Bias ^b^	Inconsistency	Indirectness	Imprecision	Publication Bias ^c^	
Survival outcomes										
OS	15	7032	2087	1.31 [1.20, 1.44]	Serious (-1)	No inconsistency	No indirectness	No imprecision	Unlikely	Very Low
OSR										
1-year	12	1030/1286	1177/1342	0.96 [0.93, 1.00]	Serious (-1)	No inconsistency	No indirectness	No imprecision	Unlikely	Very Low
2-year	12	779/1286	1006/1342	0.89 [0.84, 0.94]	Low	No inconsistency	No indirectness	No imprecision	Unlikely	Low
3-year	12	618/1286	870/1342	0.86 [0.78, 0.91]	Low	No inconsistency	No indirectness	No imprecision	Unlikely	Low
5-year	12	459/1268	723/1359	0.79 [0.72, 0.87]	Low	No inconsistency	No indirectness	No imprecision	Unlikely	Low
DFS	7	532	765	1.49 [1.24, 1.79]	Serious (-1)	No inconsistency	No indirectness	No imprecision	Unlikely	Very Low
DFSR										
1-year	6	294/422	568/670	0.87 [0.81, 0.94]	Low	No inconsistency	No indirectness	No imprecision	Unlikely	Low
2-year	6	211/422	494/670	0.76 [0.68, 0.85]	Low	No inconsistency	No indirectness	No imprecision	Unlikely	Low
3-year	6	177/422	457/670	0.71 [0.63, 0.81]	Low	No inconsistency	No indirectness	No imprecision	Unlikely	Low
5-year	5	137/371	399/632	0.71 [0.61, 0.83]	Low	Serious (-1)	No indirectness	No imprecision	Unlikely	Very Low
**Recurrence outcomes**										
Total recurrence	5	172/406	215/705	1.53 [0.82, 2.84]	Low	No inconsistency	No indirectness	No imprecision	Unlikely	Low
Local recurrence	4	50/308	77/498	0.85 [0.60, 1.20]	Low	No inconsistency	No indirectness	No imprecision	Unlikely	Low
Liver	2	13/176	10/261	1.49 [0.69, 3.21]	Low	No inconsistency	No indirectness	No imprecision	Unlikely	Low
Peritoneal	4	14/262	33/628	0.74 [0.39, 1.43]	Low	Serious (-1)	No indirectness	No imprecision	Unlikely	Very Low
Lung	1	5/85	5/82	0.96 [0.29, 3.21]	Low	No inconsistency	No indirectness	No imprecision	Unlikely	Low
Anastomosis	2	5/170	6/375	1.42 [0.46, 4.40]	Low	No inconsistency	No indirectness	No imprecision	Unlikely	Low
Lymph node	4	42/262	37/628	2.90 [1.12, 7.52]	Low	No inconsistency	No indirectness	No imprecision	Unlikely	Low
Multiple recurrence	3	34/231	27/455	3.14 [0.64, 15.52]	Low	Serious (-1)	No indirectness	No imprecision	Unlikely	Very Low
**Operative and hospitalization outcomes**										
Operative time	9	827	1045	35.75 [3.08, 68.42]	Very Serious (-2)	Very Serious (-2)	No indirectness	No imprecision	Unlikely	Very Low
Intraoperative blood loss	8	795	926	32.16 [4.83, 59.49]	Very Serious (-2)	Very Serious (-2)	No indirectness	Serious (-1)	Unlikely	Very Low
R0 resection	6	460/525	677/752	0.99 [0.95, 1.03]	Unclear	Very Serious (-2)	No indirectness	No imprecision	Unlikely	Very Low
Number of lymph node retrieved	13	1252	1453	-4.17 [-6.78, -1.56]	Low	No inconsistency	No indirectness	No imprecision	Unlikely	Low
Number of positive lymph node retrieved	10	847	1046	-0.37 [-0.74, -0.01]	Low	No inconsistency	No indirectness	Serious (-1)	Unlikely	Very Low
Hospital stay	11	990	1183	2.47 [0.60, 4.35]	Low	No inconsistency	No indirectness	No imprecision	Unlikely	Low
**Complication summary**										
Total complication	13	454/1234	373/1470	1.28 [1.15, 1.42]	Very Serious (-2)	Very Serious (-2)	No indirectness	No imprecision	Unlikely	Very Low
Complication (Claviene Dindo classification III-IV)	5	53/332	82/575	1.14 [0.83, 1.57]	Very Serious (-2)	No inconsistency	No indirectness	Serious (-1)	Unlikely	Very Low
Postoperative mortality	11	30/1140	33/1163	1.09 [0.65, 1.85]	Unclear	Very Serious (-2)	No indirectness	No imprecision	Unlikely	Very Low
**Complications**										
Postoperative haemorrhage	4	14/645	5/378	1.32 [0.54, 3.24]	Serious (-1)	No inconsistency	No indirectness	No imprecision	Unlikely	Very Low
Anastomotic bleeding	1	2/30	3/181	4.02 [0.70, 23.08]	Serious (-1)	No inconsistency	No indirectness	No imprecision	Unlikely	Very Low
Intraperitoneal bleeding	2	0/139	3/380	0.68 [0.09, 5.40]	Low	No inconsistency	No indirectness	No imprecision	Unlikely	Low
Anastomotic leakage	11	52/961	66/1339	1.58 [1.10, 2.27]	Low	No inconsistency	No indirectness	Serious (-1)	Unlikely	Very Low
Reoperation	4	19/594	14/482	1.06 [0.58, 1.95]	Low	No inconsistency	No indirectness	No imprecision	Unlikely	Low
Wound infection	2	11/200	5/378	4.01 [1.49, 10.80]	Very Serious (-2)	No inconsistency	No indirectness	No imprecision	Unlikely	Very Low
Peritonitis	7	18/823	26/833	0.81 [0.44, 1.50]	Low	No inconsistency	No indirectness	Serious (-1)	Unlikely	Very Low
Pneumothorax	1	6/140	0/60	5.62 [0.32, 98.28]	Low	No inconsistency	No indirectness	No imprecision	Unlikely	Low
Jejunal stump leakage	1	0/140	1/60	0.14 [0.01, 3.49]	Low	No inconsistency	No indirectness	No imprecision	Unlikely	Low
Duodenum stump leakage	1	0/140	3/60	0.06 [0.00, 1.18]	Low	No inconsistency	No indirectness	Serious (-1)	Unlikely	Very Low
Wound rupture	2	2/424	2/107	0.23 [0.03, 1.52]	Low	No inconsistency	No indirectness	No imprecision	Unlikely	Low
Esophago-bronchial fistula	1	0/140	1/60	0.14 [0.01, 3.49]	Low	No inconsistency	No indirectness	No imprecision	Unlikely	Low
Gastric tube perforation	1	4/140	0/60	3.89 [0.21, 71.21]	Serious (-1)	No inconsistency	No indirectness	No imprecision	Unlikely	Very Low
Necrosis of gastric tube	1	1/140	0/60	1.30 [0.05, 31.41]	Low	No inconsistency	No indirectness	Serious (-1)	Unlikely	Very Low
Pancreatic fistula	3	15/306	13/219	1.02 [0.53, 1.98]	Low	No inconsistency	No indirectness	No imprecision	Unlikely	Low
Pneumonia	8	100/820	101/959	1.71 [1.30, 2.25]	Low	No inconsistency	No indirectness	No imprecision	Unlikely	Low
Pleural effusion	3	17/222	25/395	1.92 [1.04, 3.52]	Serious (-1)	No inconsistency	No indirectness	No imprecision	Unlikely	Very Low
Gastroparesis	1	2/109	4/199	0.91 [0.17, 4.90]	Serious (-1)	No inconsistency	No indirectness	No imprecision	Unlikely	Very Low
Anastomotic stricture	2	11/172	46/313	0.78 [0.43, 1.41]	Low	No inconsistency	No indirectness	No imprecision	Unlikely	Low
Pyothorax	1	4/85	1/82	3.86 [0.44, 33.80]	Low	No inconsistency	No indirectness	No imprecision	Unlikely	Low
Bowel obstruction	1	2/284	1/47	0.33 [0.03, 3.58]	Very Serious (-2)	Very Serious (-2)	No indirectness	Serious (-1)	Unlikely	Very Low
Dumping syndrome	1	1/32	5/119	0.74 [0.09, 6.14]	Unclear	Very Serious (-2)	No indirectness	No imprecision	Unlikely	Very Low

*CI *Confidence interval, *DFS *Disease-free survival, *DFSR *Disease-free survival rate, *GRADE *Grading of Recommendations Assessment, Development and Evaluation, *OS *Overall survival, *OSR *Overall survival rate

^a^ Differences: hazard ratio (HR) for OS and DFS; risk ratios (RR) for OSR, DFSR, recurrence outcomes, operative and hospitalization outcomes, complication summary and complications

^b^ Risk of bias assessed using the Newcastle-Ottawa Scale (NOS) for non-randomized studies and Jadad scale for randomized controlled trials

^c^ Publication bias was assessed by Egger’s and Begg’s tests

### Survival

**Fig. 2:**
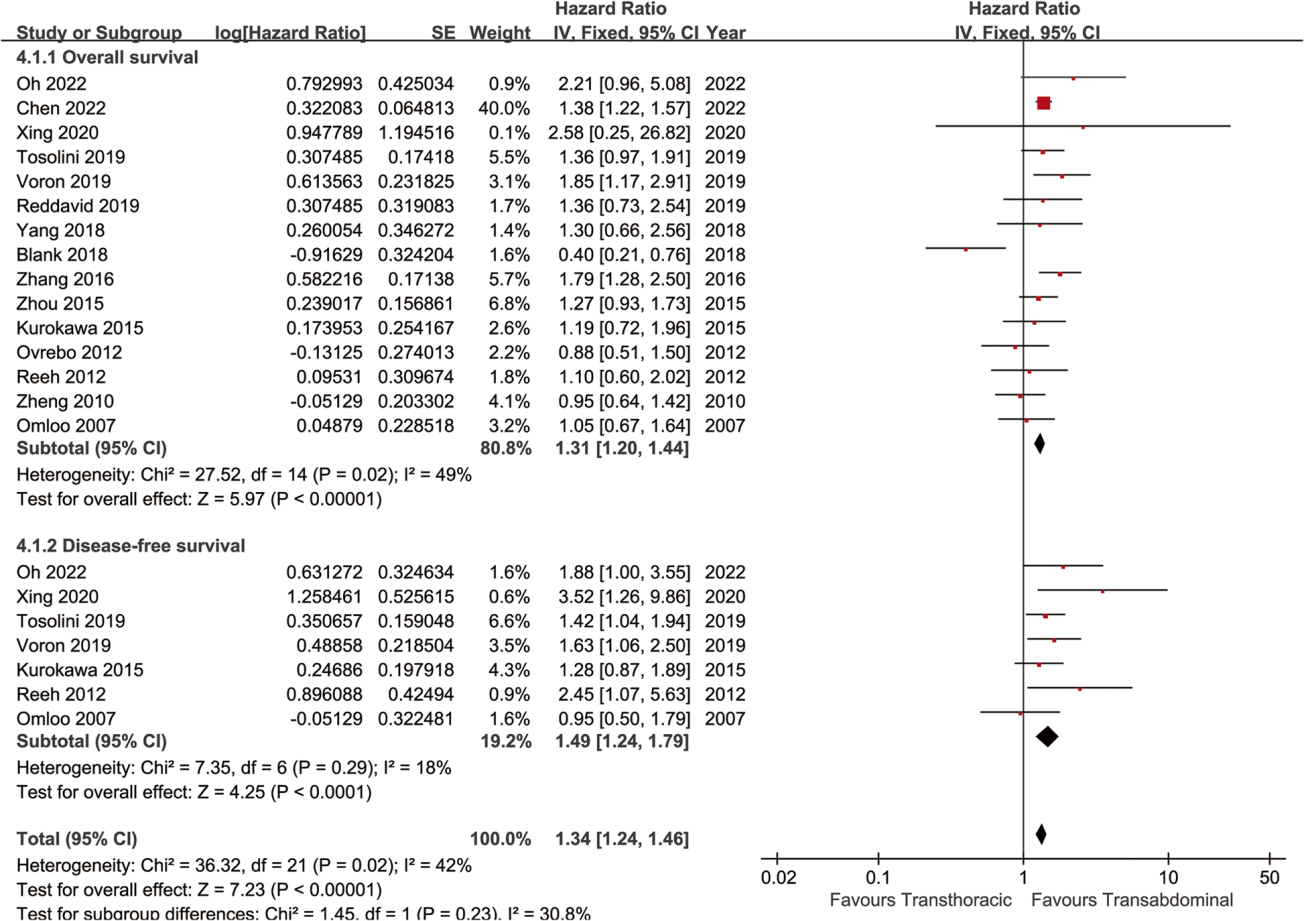
Forest plots of overall survival and disease-free survival associated with transthoracic surgery and transabdominal surgery

**Fig. 3:**
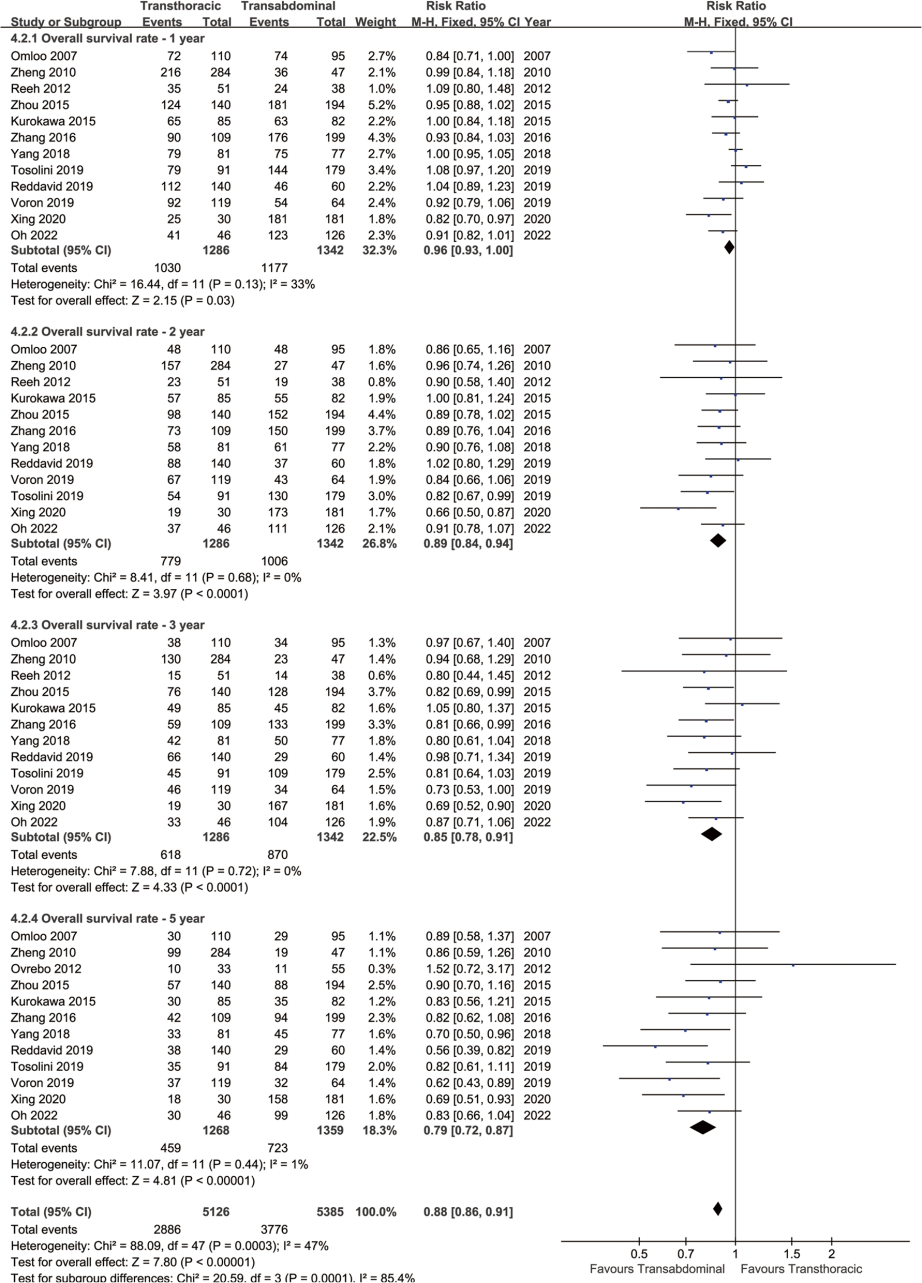
Forest plots of overall survival rate at 1, 2, 3, 5 years associated with transthoracic surgery and transabdominal surgery

**Fig. 4:**
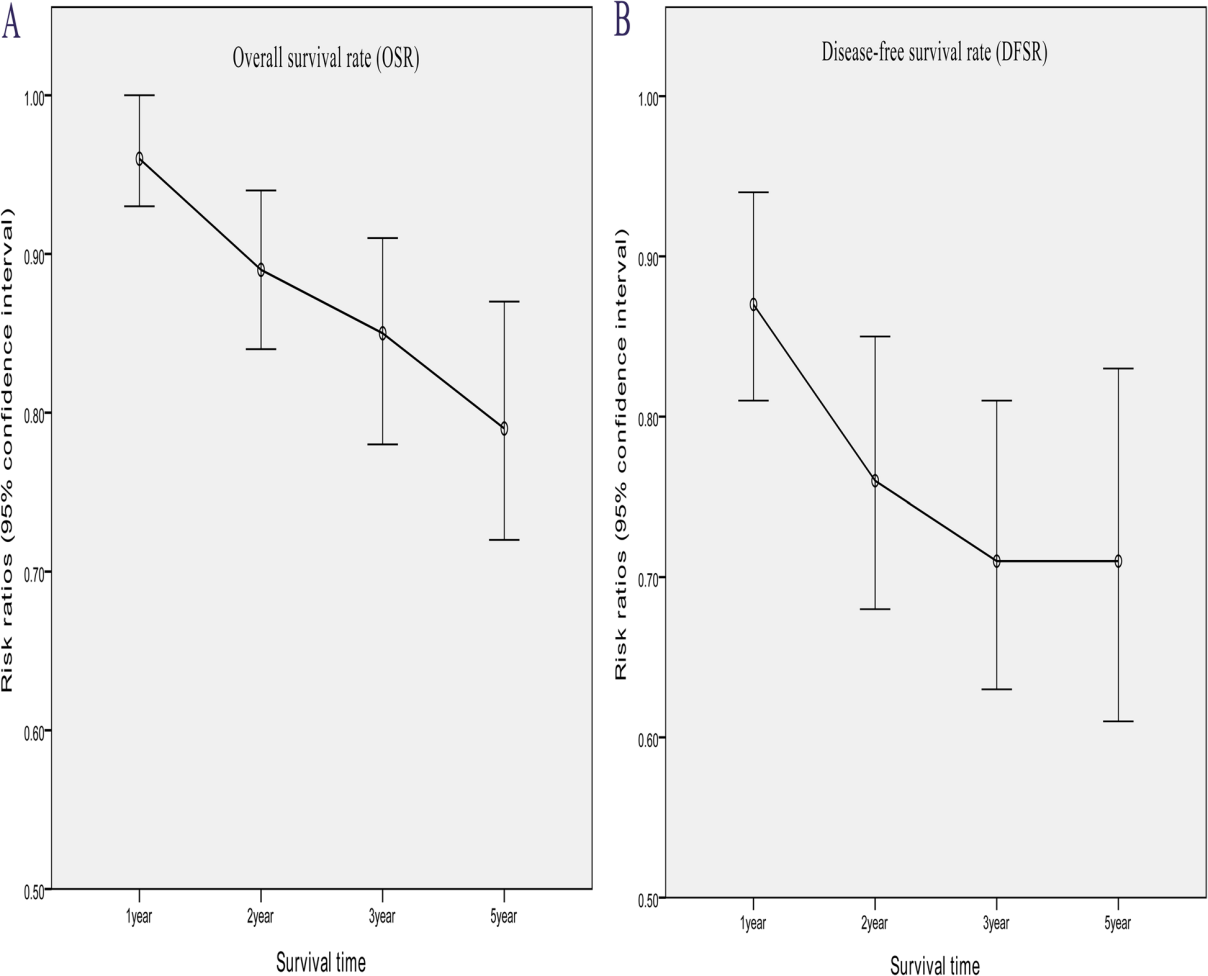
Line charts of overall survival rate (1, 2, 3, 5 years, A) and disease-free survival rate (1, 2, 3, 5 years, B) associated with transthoracic surgery and transabdominal surgery

Longer OS was achieved in the TA group (HR: 1.31 [1.20 ~ 1.44], *p* < 0.00001, Fig. 2). In the analysis of OSR, the survival rate of the TA group was higher than that of the TT group in all years (1, 2, 3, and 5 years) (Fig. 3). The survival advantage of OSR increased with time (RR increased from 0.96 to 0.79) (Fig. 4A).

**Fig. 5:**
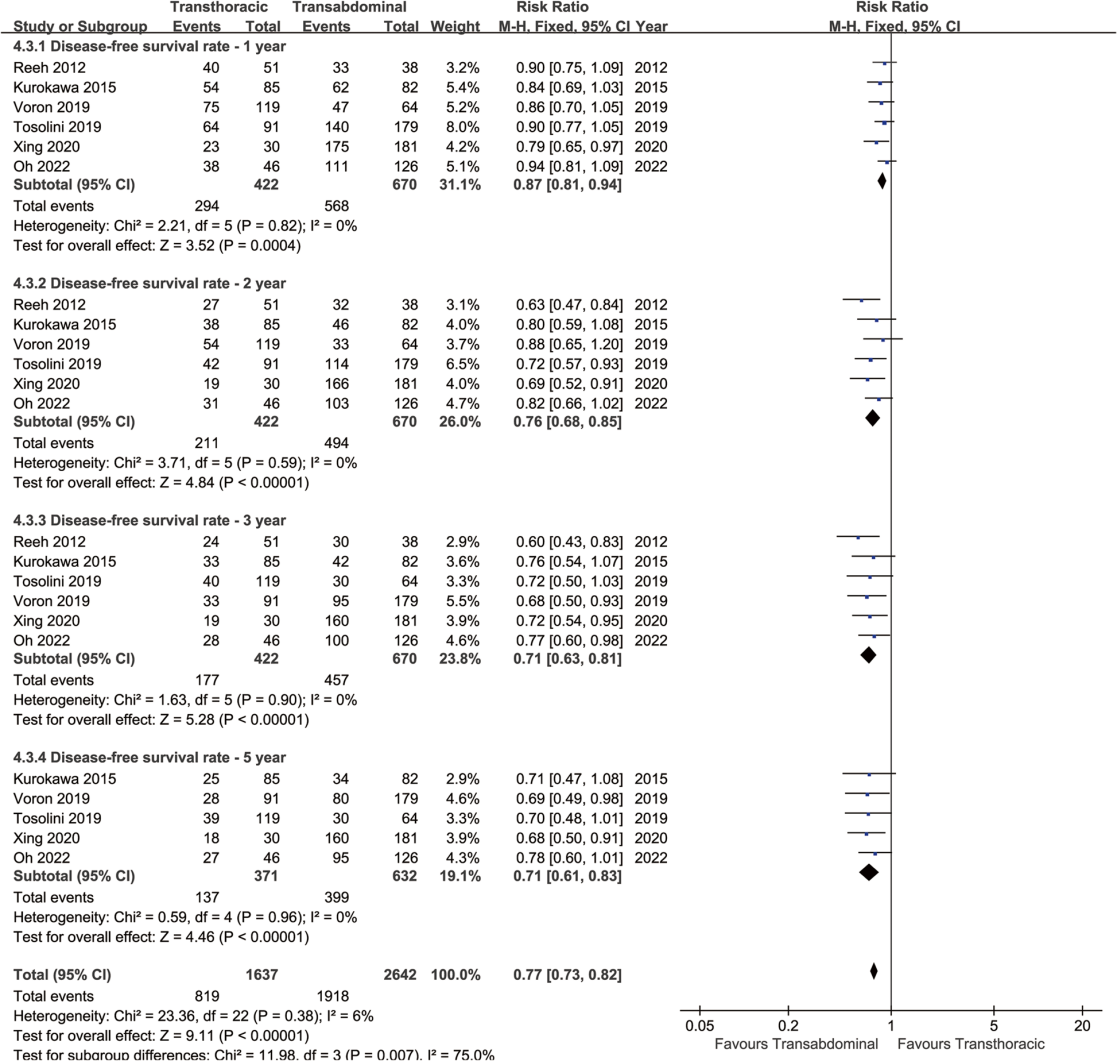
Forest plots of disease-free survival rate at 1, 2, 3, 5 years associated with transthoracic surgery and transabdominal surgery

Longer DFS was achieved in the TA group (HR: 1.49 [1.24 ~ 1.79], *p* < 0.0001, Fig. 2). In the analysis of DFSR, the survival rate of the TA group was higher than that of the TT group in all years (1, 2, 3, and 5 years) (Fig. 5). The survival advantage of DFSR increased with time (RR increased from 0.87 to 0.71) (Fig. 4B).

#### Subgroup analysis of survival

A subgroup analysis of OS and DFS was performed according to publication year (earlier than 2017 or 2017–2023), region (East Asia or Europe), TT group (thoracoabdominal or left transthoracic), surgical volume (> 20 per year or < 20 per year), and study design (RCT or CT). In the analysis of OS and DFS, there was no change in the preferred procedure among all subgroups. However, in the subgroups of region (Europe), TT group (left transthoracic), surgical volume (< 20 per year), and study design (RCT), there was no significant difference in OS associated with TA procedures. In subgroups of surgical volume (> 20 per year) and study design (RCT), there was no significant difference in the DFS advantage of the TA group (Table 3).

**Table 3:** Subgroup analysis of overall survival and disease-free survival

Subgroups	No.of studies	Overall Survival		No.of studies	Disease-Free Survival	
		HR (95% CI)	*P*		HR (95% CI)	*P*
**Total**	15	1.31 (1.20–1.44)	< 0.00001	7	1.49 (1.24–1.79)	< 0.0001
**Published year**						
Earlier than 2017	8	1.36 (1.22–1.52)	< 0.00001	4	1.60 (1.27–2.01)	< 0.0001
2017–2023	7	1.22 (1.04–1.43)	0.01	3	1.30 (0.96–1.77)	0.09
**Region**						
East Asia	8	1.37 (1.23–1.51)	< 0.00001	3	1.55 (1.13–2.12)	0.007
Europe	7	1.08 (0.79–1.49)	0.63	4	1.46 (1.16–1.83)	0.001
**TT group** ^**a**^						
TT group 1	5	1.39 (1.25–1.55)	< 0.00001	1	1.28 (0.87–1.89)	0.21
TT group 2	10	1.13 (0.87–1.48)	0.36	6	1.55 (1.26–1.91)	< 0.0001
**Surgical volume (included in the study)**						
>20 per year	9	1.35 (1.23–1.49)	< 0.00001	5	1.41 (1.14–1.74)	0.001
<20 per year	6	1.05 (0.69–1.61)	0.81	2	1.77 (1.21–2.60)	0.003
**Study design**						
RCT	2	1.11 (0.68–1.72)	0.75	2	1.18 (0.85–1.64)	0.33
CT	13	1.26 (1.06–1.51)	0.01	5	1.65 (1.32–2.06)	< 0.00001

*HR *Hazard ratio, *CI *Confidence interval, *TA *Transabdominal, *TT *Transthoracic, *RCT *Randomized controlled trial, *CT *Cohort study

^a^ TT group was divided into two groups:1. TT group 1: Left intercostal thoracotomy approach; 2. TT group 2: Right intercostal thoracotomy approach + median laparotomy

### Intraoperative and hospitalization indicators

**Fig. 6:**
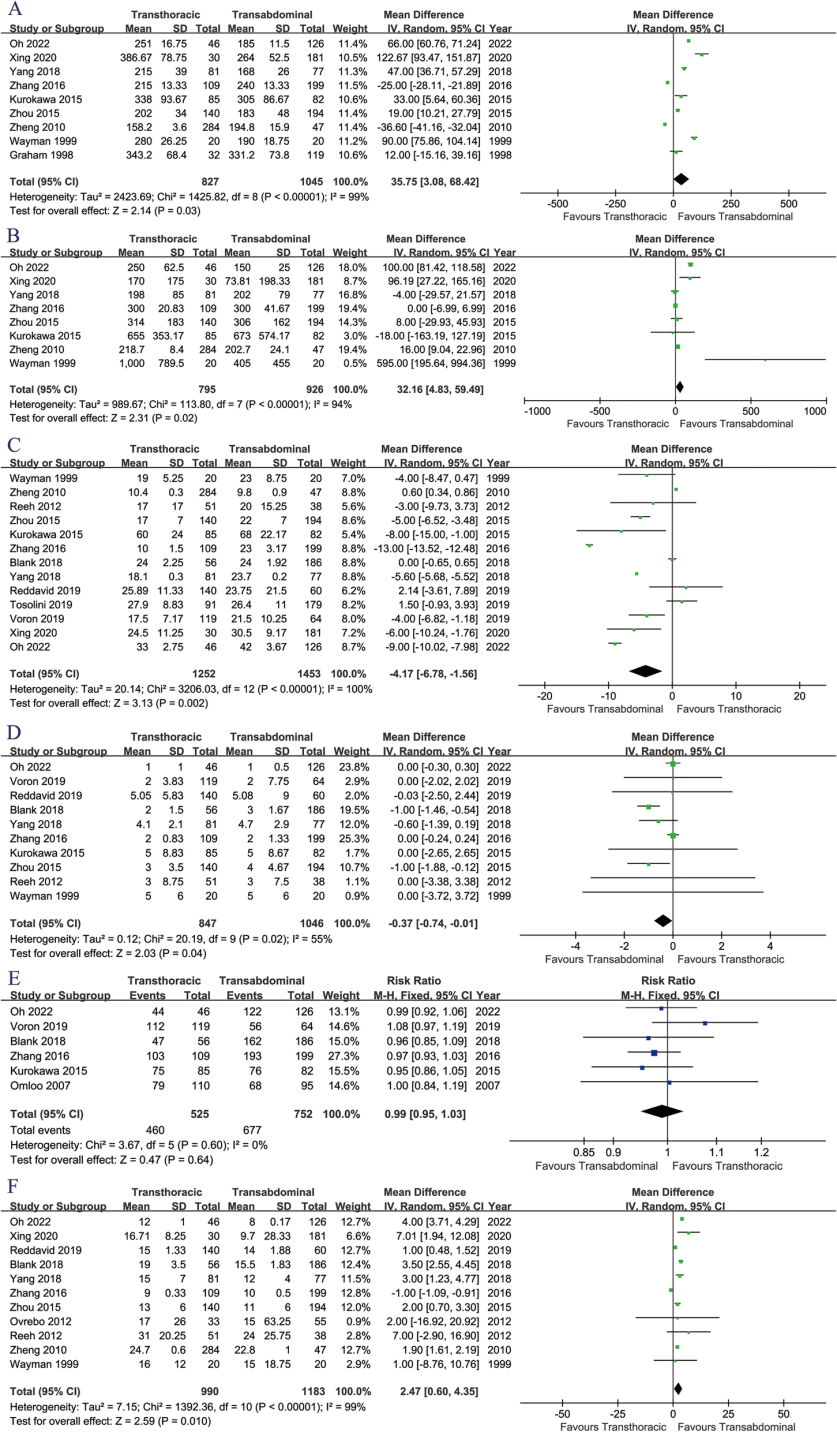
Forest plots of intraoperative and hospitalization indicators associated with transthoracic surgery and transabdominal surgery: (A) operating time; (B) intraoperative blood loss; (C) number of lymph node retrieved; (D) number of positive lymph node retrieved; (E) R0 resection; (F) hospital stay

Operating time (MD: 35.75 [3.08 ~ 68.42] minutes, *p* = 0.03, Fig. 6A), intraoperative blood loss (MD: 32.16 [4.83 ~ 59.49] mL, *p* = 0.02, Fig. 6B), number of lymph nodes retrieved (MD: -4.17 [4.83 ~ 59.49], *p* = 0.02, Fig. 6C), number of positive lymph nodes retrieved (MD: -3.07 [-0.74~-0.01], *p* = 0.04, Fig. 6D), and length of hospital stay (MD: 2.47 [0.60 ~ 4.35] days, *p* = 0.01, Fig. 6F) were better in the TA group. The R0 resection rate (RR: 0.99 [0.95 ~ 1.03], *p* = 0.64, Fig. 6E) was similar between the two groups.

### Recurrence

Fewer lymph node recurrences (RR: 2.90 [1.12 ~ 7.52], *p* = 0.03**)** were found in the TA group. The total recurrence, local recurrence, liver recurrence, peritoneal recurrence, lung recurrence, anastomosis recurrence and multiple recurrence rates were similar between the two groups (Figure S1).

### Complications

**Fig. 7:**
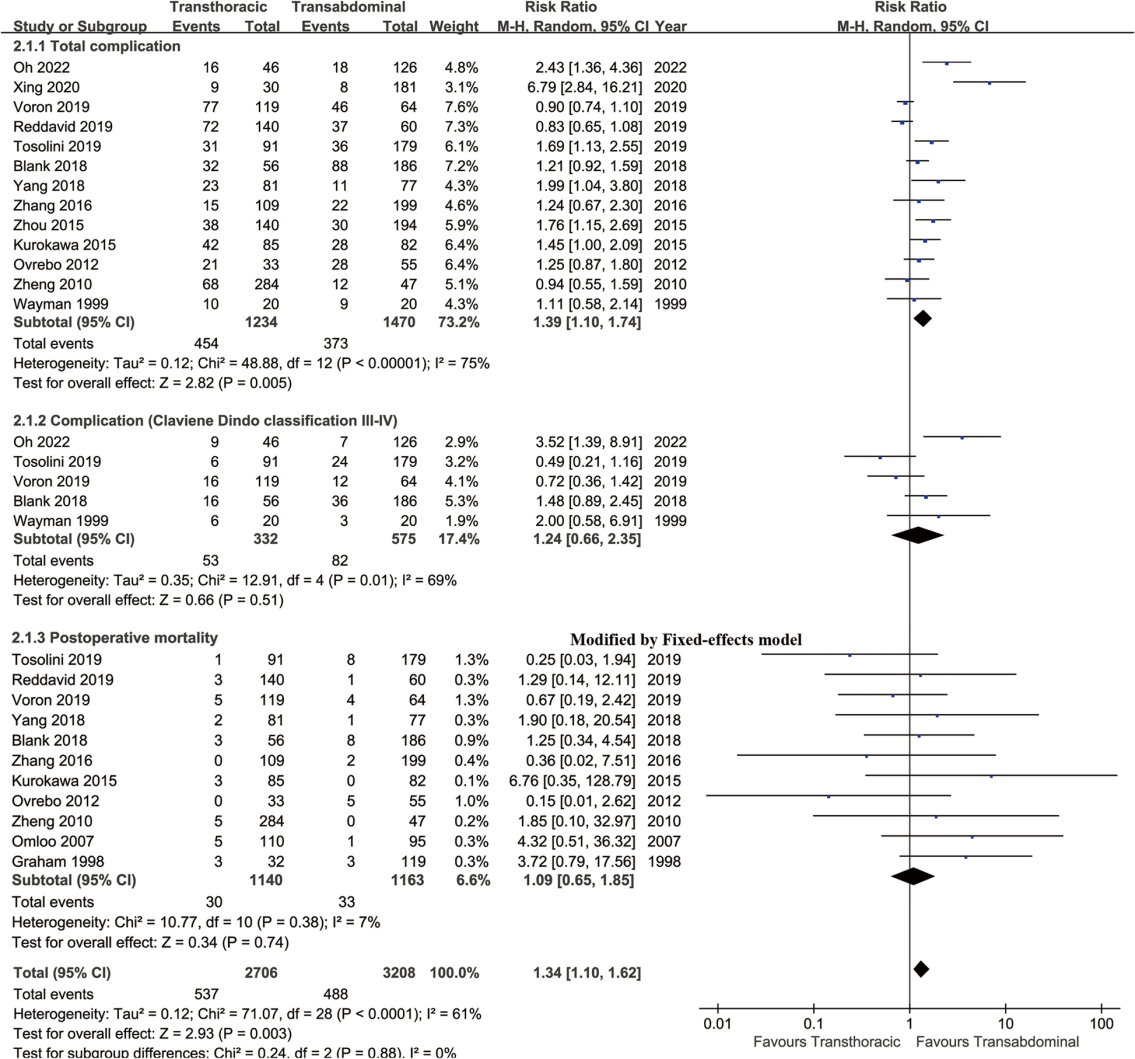
Forest plots of complication summary (total complication, complication [Claviene Dindo classification III-IV] and postoperative mortality) associated with transthoracic surgery and transabdominal surgery

In summary, more total complications (RR: 1.39 [1.10 ~ 1.74], *p* = 0.005) were found in the TA group. Complications (Clavien‒Dindo classification III–-IV) and postoperative mortality were similar between the two groups (Fig. 7).

Fewer anastomotic leakages (RR: 1.58 [1.10 ~ 2.27], *p* = 0.01), pneumonia (RR: 1.71 [1.30 ~ 2.25], *p* = 0.0001) and pleural effusion (RR: 1.92 [1.04 ~ 3.52], *p* = 0.04) were found in the TA group. Similar incidences of postoperative hemorrhage, anastomotic bleeding, intraperitoneal bleeding, anastomotic leakage, reoperation, wound infection, peritonitis, pneumothorax, jejunal stump leakage, duodenum stump leakage, wound rupture, esophago-bronchial fistula, gastric tube perforation, necrosis of gastric tube, pancreatic fistula, gastroparesis, anastomotic stricture, pyothorax, bowel obstruction and dumping syndrome were found between the two groups (Table 4, Figure S2).

**Table 4:** Complications in TT group and TA group

Complication	Studies involved	TT group		TA group		Total incidence	Risk ratio	P
		Event/total	%	Event/total	%			
**Complication summary**								
Total complication	13	454/1234	36.79%	373/1470	25.37%	30.58%	1.28 [1.15, 1.42]	0.005
Complication (Claviene Dindo classification III-IV)	5	53/332	15.96%	82/575	14.26%	14.88%	1.14 [0.83, 1.57]	0.51
Postoperative mortality	11	30/1140	2.63%	33/1163	2.84%	2.74%	1.09 [0.65, 1.85]	0.74
**Complications**								
Anastomotic stricture	2	11/172	6.40%	46/313	14.70%	11.75%	0.78 [0.43, 1.41]	0.4
Pneumonia	8	100/820	12.20%	101/959	10.53%	11.30%	1.71 [1.30, 2.25]	0.0001
Pleural effusion	3	17/222	7.66%	25/395	6.33%	6.81%	1.92 [1.04, 3.52]	0.04
Pancreatic fistula	3	15/306	4.90%	13/219	5.94%	5.33%	1.02 [0.53, 1.98]	0.95
Anastomotic leakage	11	52/961	5.41%	68/1339	5.08%	5.22%	1.58 [1.10, 2.27]	0.01
Dumping syndrome	1	1/32	3.13%	5/119	4.20%	3.97%	0.74 [0.09, 6.14]	0.78
Reoperation	4	19/594	3.20%	14/482	2.90%	3.07%	1.06 [0.58, 1.95]	0.65
Pneumothorax	1	6/140	4.29%	0/60	0.00%	3.00%	5.62 [0.32, 98.28]	0.24
Pyothorax	1	4/85	4.71%	1/82	1.22%	2.99%	3.86 [0.44, 33.80]	0.22
Wound infection	2	11/200	5.50%	5/378	1.32%	2.77%	4.01 [1.49, 10.80]	0.58
Peritonitis	7	18/823	2.19%	26/833	3.12%	2.66%	0.81 [0.44, 1.50]	0.5
Anastomotic bleeding	1	2/30	6.67%	3/181	1.66%	2.37%	4.02 [0.70, 23.08]	0.12
Gastric tube perforation	1	4/140	2.86%	0/60	0.00%	2.00%	3.89 [0.21, 71.21]	0.36
Gastroparesis	1	2/109	1.83%	4/199	2.01%	1.95%	0.91 [0.17, 4.90]	0.92
Postoperative haemorrhage	4	14/645	2.17%	5/378	1.32%	1.86%	1.32 [0.54, 3.24]	0.68
Duodenum stump leakage	1	0/140	0.00%	3/60	5.00%	1.50%	0.06 [0.00, 1.18]	0.06
Bowel obstruction	1	2/284	0.70%	1/47	2.13%	0.91%	0.33 [0.03, 3.58]	0.36
Wound rupture	2	2/424	0.47%	2/107	1.87%	0.75%	0.23 [0.03, 1.52]	0.13
Intraperitoneal bleeding	2	0/139	0.00%	3/380	0.79%	0.58%	0.68 [0.09, 5.40]	0.85
Jejunal stump leakage	1	0/140	0.00%	1/60	1.67%	0.50%	0.14 [0.01, 3.49]	0.23
Esophago-bronchial fistula	1	0/140	0.00%	1/60	1.67%	0.50%	0.14 [0.01, 3.49]	0.23
Necrosis of gastric tube	1	1/140	0.71%	0/60	0.00%	0.50%	1.30 [0.05, 31.41]	0.87

*TA *Transabdominal, *TT *Transthoracic

### Sensitivity analysis

In the analysis of intraoperative blood loss, operating time, number of lymph nodes retrieved, number of positive lymph nodes retrieved, and length of hospital stay, significant heterogeneity was found. After removal of each study, the tendency of the results did not change, which confirmed the stability and reliability of these results (Figure S3).

### Publication bias

Funnel plots based on the data regarding survival (OS, PFS) (Figure S4A), OSR (Figure S4B), and DFSR (Figure S4C) suggested that there was no significant publication bias.

## Discussion

EGJA is one of the major cancers with high morbidity and mortality rates worldwide; however, its treatment is not standardized, and the therapeutic effect is unsatisfactory [27]. Whether a transthoracic (TT) procedure by a thoracic surgeon or a transabdominal (TA) by a gastrointestinal surgeon is best for Siewert type II esophagogastric junction adenocarcinoma (EGJA) remains unknown [8, 12, 13]. We first conducted this meta-analysis to answer this question. In this study, the TA procedure achieved longer OS and DFS than the TT procedure. The OSR and the DFSR increased with time. More total/positive lymph nodes were retrieved, and fewer lymph node recurrences were found in the TA group. In the analysis of perioperative outcomes, a longer operating time, more intraoperative blood loss, and a longer hospital stay were found in the TA group. In the analysis of complications, more total complications, anastomotic leakage, pneumonia, and pleural effusion were found in the TT group.

In this analysis, longer OS and DFS were the strongest supporting evidence for the TA group. Better survival results were also reported by Voron et al.’s and Xing et al.’s studies [7, 8]. Two results in our study might explain this advantage: (1) More total lymph nodes and positive lymph nodes were retrieved in the TA group, which directly led to a lower rate of lymph node recurrence after surgery. We believed that the insufficient dissection of lymph nodes is mainly related to the increased difficulty of abdominal lymph node dissection in TT procedures and thoracic surgeons’ lack of understanding of abdominal lymph node dissection [9, 10, 28]. (2) Another explanation for this advantage is the safety of the surgery. In our analysis, a longer operating time, more intraoperative blood loss, a longer hospital stay, and more complications were found in the TT group, which directly led to the higher perioperative mortality rate and indirectly affected the long-term survival of patients [11]. In subgroup analysis of survival, TA procedures remained the preferred choice among all subgroups. The OSR (RR increased from 0.96 to 0.79) and DFSR (RR increased from 0.87 to 0.71) increased with time. In summary, we believe that TA procedures had survival advantages over TT procedures.

Fewer postoperative complication was another advantage of the TA approach. The addition of thoracotomy and thoracic lymph node dissection will increase the incidence of complications, which is also in line with the actual clinical situation. In our study, more total complications, anastomotic leakages, pneumonia cases, and pleural effusion cases were found in the TT group. Anastomotic leakage is the most troublesome complication after the resection of esophageal and cardiac tumors and one of the main causes for perioperative death. In our study, the probability of anastomotic leakage was 5.41% in the TT group and 5.08% in the TA group; the tendency of favoring TA was confirmed in the 8/11 relevant studies [8, 12, 13, 19, 20, 22, 23, 25]. Xing et al. reported a similar result and suggested that a prolonged operation and difficult reinforcement of the anastomosis might be the cause of this difference [8]. We believe that better blood supply and higher probability of incarceration of the cardiac hole may also explain the higher probability of anastomotic leakage in the TT group. Pneumonia is highly prevalent in patients who undergo the TT approach and may endanger the patient’s life during the perioperative period. The tendency to favor TA was confirmed in all 8 relevant studies [9, 11, 12, 19, 20, 22, 23, 26]. The higher rate of pneumonia in the TT group was mainly due to a chest wall injury caused by thoracotomy and the collapse and expansion of the lung during operation [29]. Based on the above reasons, for the EGJA patients in poor physical condition who cannot tolerate thoracotomy, the TA surgery is a good choice.

Although this study systematically analyzed all relevant studies with large size samples and all the involved outcomes, there were still some deficiencies that need to be considered. First, not all the included studies (2/17) were RCTs, which might decrease the evidence level of the results. Second, there were differences in the surgical volume, surgical methods, and criteria for determining the outcomes in different research centers, especially in the TT group. Although subgroup analysis and sensitivity analysis were conducted, there was still heterogeneity in the combined analysis of the outcomes. Third, some meta-analyses involved relatively few or even only one study, which may have affected the reliability of the results. Fourth, the patients were enrolled at different time points in these 17 studies, possibly leading to major changes in surgical methods and surgical requirements that might affect the consistency of outcomes. Fifth, due to insufficient data provided, there might be differences between the two groups regarding tumor size, staging, and lymph node metastasis, which might affect the comparability of the data between the groups.

## Conclusion

For patients with Siewert type II EGJA, the TA procedure was a better choice because of its ability to prolong OS and DFS when compared with the TT procedure. The OSR and DFSR increased with time. More complete lymph node dissection and fewer lymph node recurrences were the main reasons for the survival advantage seen in the TA group. In terms of safety, more total complications, anastomotic leakages, pneumonia cases, and pleural effusion cases were found in the TT group. However, due to the above deficiencies, the conclusions of this study still need to be verified in large sample RCTs in the future.

### Supplementary Information

**Additional file 1: Figure S1 **Forest plots of recurrences associated with transthoracic surgery and transabdominal surgery. **Figure S2 **Forest plots of complications associated with transthoracic surgery and transabdominal surgery. **Figure S3**Sensitivity analysis ofintraoperative blood loss(A), operating time(B), number of lymph node retrieved(C), number of positive lymph node retrieved (D) and hospital stay(E).** Figure S4 **Funnel plots of survival (overallsurvivalanddisease-free survival, A), overallsurvivalrate (1, 2, 3, 5 years, B) and disease-free survival rate (1, 2, 3, 5 years, C). **Table S1**PRISMA 2009 Checklist. **Table S2 **Search strategy. **Table S3 **Methodological quality assessments of the included studies.

## Data Availability

The data sets used and/or analysed during the current study are available from the corresponding author on reasonable request.

## Abbreviations

CT: Cohort study
CI: Confidence interval
DFS: Disease-free survival
DFSR: Disease-free survival rate
EGJA: Esophagogastric junction adenocarcinoma
GRADE: Grading of Recommendations Assessment, Development and Evaluation
HR: Hazard ratio
MeSH: Medical subject headings
MD: Mean difference
NOS: Newcastle-Ottawa Scale
OS: Overall survival
OSR: Overall survival rate
PRISMA: Preferred Reporting Items for Systematic Reviews and Meta-Analyses
RCT: Randomized clinical trial
RR: Risk ratio
TA: Transabdominal
TNM: Tumor Node Metastasis
TT: Transthoracic
