# Supplementary material for: Effect of different surgical approaches on the survival and safety of Siewert type II esophagogastric junction adenocarcinoma: a systematic review and meta-analysis
Source: BMC Cancer. 2023 Nov 21;23:1130. doi: 10.1186/s12885-023-11640-5 (PMC10662530; doi:10.1186/s12885-023-11640-5)
Supplement: Supplementary file 1 — Additional file 1: Figure S1 Forest plots of recurrences associated with transthoracic surgery and transabdominal surgery. Figure S2 Forest plots of complications associated with transthoracic surgery and transabdominal surgery. Figure S3Sensitivity analysis ofintraoperative blood loss(A), operating time(B), number of lymph node retrieved(C), number of positive lymph node retrieved (D) and hospital stay(E). Figure S4 Funnel plots of survival (overallsurvivalanddisease-free survival, A), overallsurvivalrate (1, 2, 3, 5 years, B) and disease-free survival rate (1, 2, 3, 5 years, C). Table S1PRISMA 2009 Checklist. Table S2 Search strategy. Table S3 Methodological quality assessments of the included studies. [file 12885_2023_11640_MOESM1_ESM.pdf]

Supplementary Material

Figure S1 Forest plots of recurrences associated with transthoracic surgery and transabdominal surgery.

Figure S2 Forest plots of complications associated with transthoracic surgery and transabdominal surgery.

Figure S3 Sensitivity analysis of intraoperative blood loss (A), operating time (B), number of lymph node retrieved (C), number of positive lymph node retrieved (D) and hospital stay(E).

Figure S4 Funnel plots of survival (overall survival and disease-free survival, A), overall survival rate (1, 2, 3, 5 years, B) and disease-free survival rate (1, 2, 3, 5 years, C).

Table S1 PRISMA 2009 Checklist.

Table S2 Search strategy.

Table S3 Methodological quality assessments of the included studies.

Figure S1 Forest plots of recurrences associated with transthoracic surgery and transabdominal surgery.

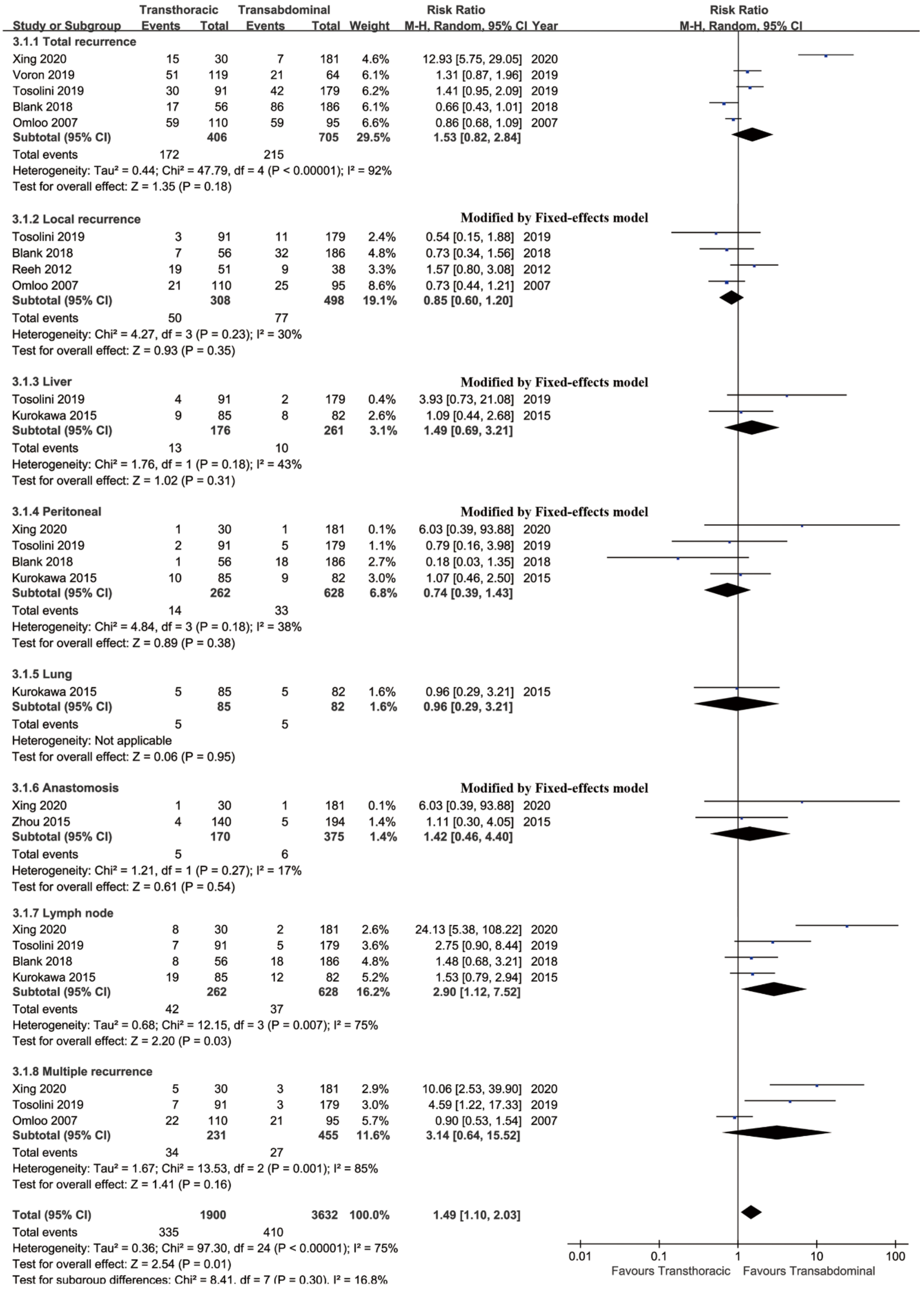

Figure S2 Forest plots of complications associated with transthoracic surgery and transabdominal surgery.

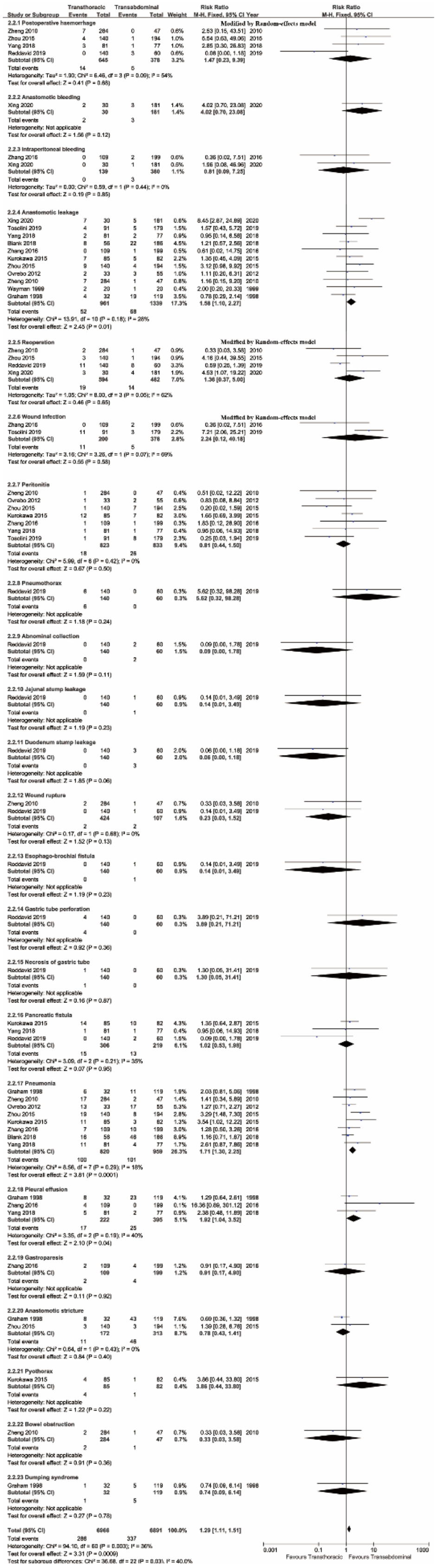

Figure S3 Sensitivity analysis of intraoperative blood loss (A), operating time (B), number of lymph node retrieved (C), number of positive lymph node retrieved (D) and hospital stay(E).

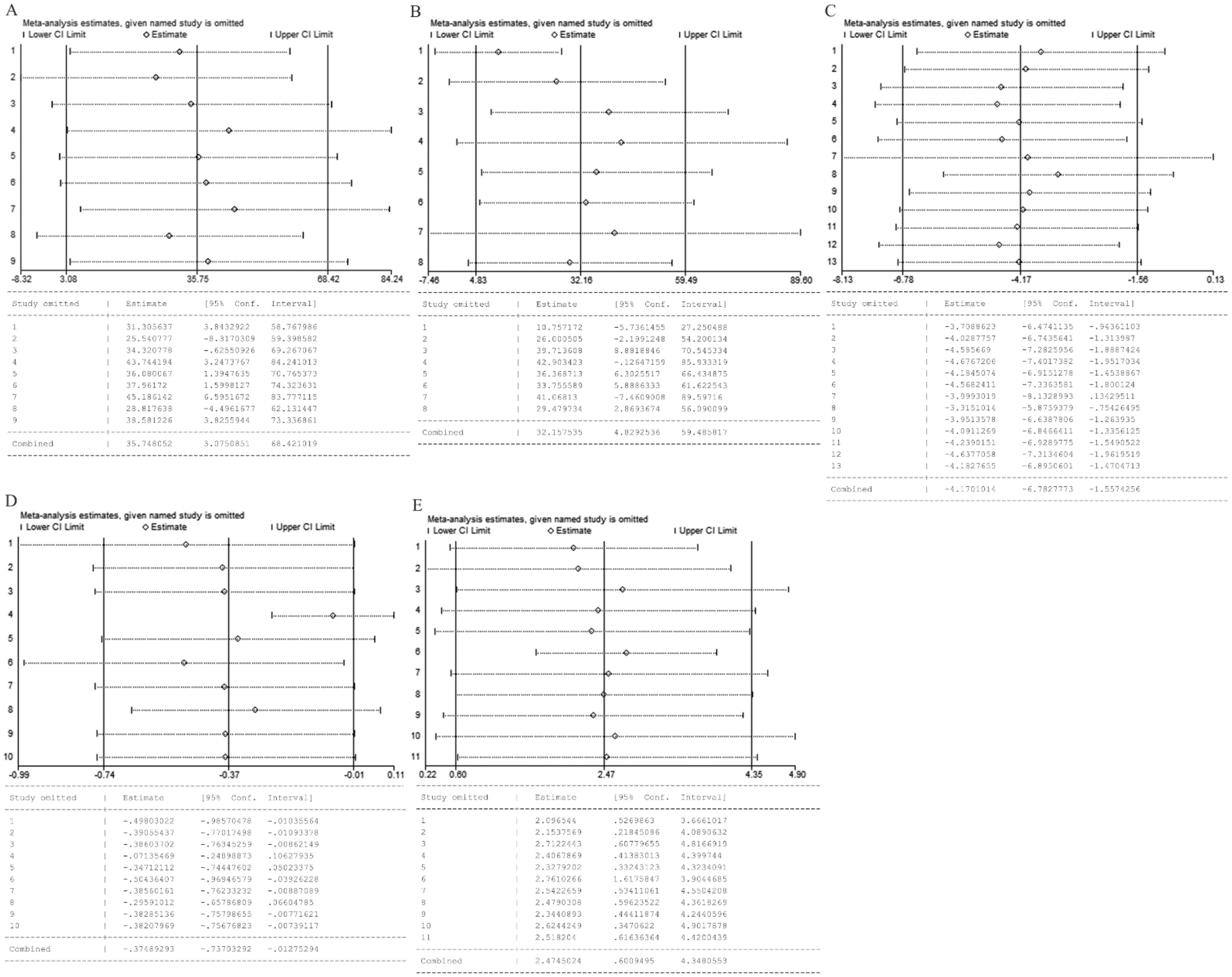

Figure S4 Funnel plots of survival (overall survival and disease-free survival, A), overall survival rate (1, 2, 3, 5 years, B) and disease-free survival rate (1, 2, 3, 5 years, C).

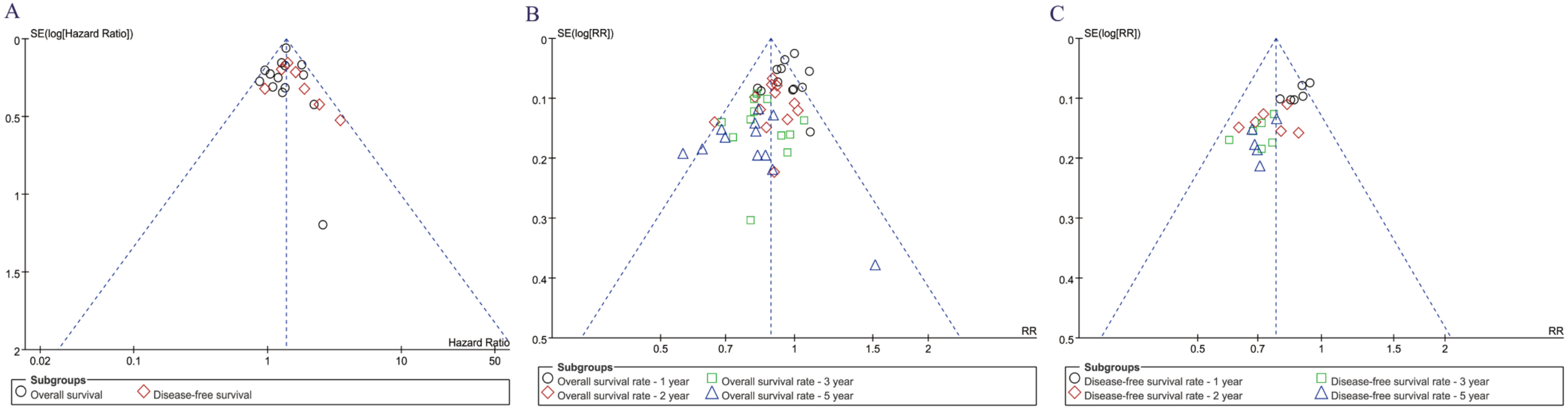

Table S1 PRISMA 2009 Checklist.

Section/topic	#	Checklist item	Reported on page #
**TITLE**			1
Title	1	Identify the report as a systematic review, meta-analysis, or both.	1
**ABSTRACT**			2-3
Structured summary	2	Provide a structured summary including, as applicable: background; objectives; data sources; study eligibility criteria, participants, and interventions; study appraisal and synthesis methods; results; limitations; conclusions and implications of key findings; systematic review registration number.	2-3
**INTRODUCTION**			4-5
Rationale	3	Describe the rationale for the review in the context of what is already known.	4-5
Objectives	4	Provide an explicit statement of questions being addressed with reference to participants, interventions, comparisons, outcomes, and study design (PICOS).	5
**METHODS**			5-8
Protocol and registration	5	Indicate if a review protocol exists, if and where it can be accessed (e.g., Web address), and, if available, provide registration information including registration number.	5
Eligibility criteria	6	Specify study characteristics (e.g., PICOS, length of follow-up) and report characteristics (e.g., years considered, language, publication status) used as criteria for eligibility, giving rationale.	5
Information sources	7	Describe all information sources (e.g., databases with dates of coverage, contact with study authors to identify additional studies) in the search and date last searched.	5-6
Search	8	Present full electronic search strategy for at least one database, including any limits used, such that it could be repeated.	5-6
Study selection	9	State the process for selecting studies (i.e., screening, eligibility, included in systematic review, and, if applicable, included in the meta-analysis).	6
Data collection process	10	Describe method of data extraction from reports (e.g., piloted forms, independently, in duplicate) and any processes for obtaining and confirming data from investigators.	6
Data items	11	List and define all variables for which data were sought (e.g., PICOS, funding sources) and any assumptions and simplifications made.	6
Risk of bias in individual studies	12	Describe methods used for assessing risk of bias of individual studies (including specification of whether this was done at the study or outcome level), and how this information is to be used in any data synthesis.	7
Summary measures	13	State the principal summary measures (e.g., risk ratio, difference in means).	7-8

Synthesis of results	14	Describe the methods of handling data and combining results of studies, if done, including measures of consistency (e.g., I^2) for each meta-analysis.	8
**Section/topic**	**#**	**Checklist item**	**Reported on page #**
Risk of bias across studies	15	Specify any assessment of risk of bias that may affect the cumulative evidence (e.g., publication bias, selective reporting within studies).	8
Additional analyses	16	Describe methods of additional analyses (e.g., sensitivity or subgroup analyses, meta-regression), if done, indicating which were pre-specified.	8
**RESULTS**			8-11
Study selection	17	Give numbers of studies screened, assessed for eligibility, and included in the review, with reasons for exclusions at each stage, ideally with a flow diagram.	8
Study characteristics	18	For each study, present characteristics for which data were extracted (e.g., study size, PICOS, follow-up period) and provide the citations.	8
Risk of bias within studies	19	Present data on risk of bias of each study and, if available, any outcome level assessment (see item 12).	8
Results of individual studies	20	For all outcomes considered (benefits or harms), present, for each study: (a) simple summary data for each intervention group (b) effect estimates and confidence intervals, ideally with a forest plot.	8-11
Synthesis of results	21	Present results of each meta-analysis done, including confidence intervals and measures of consistency.	8-11
Risk of bias across studies	22	Present results of any assessment of risk of bias across studies (see Item 15).	11
Additional analysis	23	Give results of additional analyses, if done (e.g., sensitivity or subgroup analyses, meta-regression [see Item 16]).	11
**DISCUSSION**			11-15
Summary of evidence	24	Summarize the main findings including the strength of evidence for each main outcome; consider their relevance to key groups (e.g., healthcare providers, users, and policy makers).	11-14
Limitations	25	Discuss limitations at study and outcome level (e.g., risk of bias), and at review-level (e.g., incomplete retrieval of identified research, reporting bias).	14
Conclusions	26	Provide a general interpretation of the results in the context of other evidence, and implications for future research.	14-15
**FUNDING**			15
Funding	27	Describe sources of funding for the systematic review and other support (e.g., supply of data); role of funders for the systematic review.	15

From: Moher D, Liberati A, Tetzlaff J, Altman DG, The PRISMA Group (2009). Preferred Reporting Items for Systematic Reviews and Meta-Analyses: The PRISMA Statement. PLoS Med 6(7): e1000097. doi:10.1371/journal.pmed1000097

Table S2 Search strategy.

**PubMed**<br>The database was searched on January 1, 2023, n=57.<br>Search Strategy:(Transthoracic [Title/Abstract] OR Thoracoabdominal [Title/Abstract]) AND (Transabdominal [Title/Abstract] OR Transhiatal [Title/Abstract]) AND ((Esophagogastric junction adenocarcinoma[Title/Abstract] OR Adenocarcinoma of the esophagogastric junction [Title/Abstract] OR Cardiac adenocarcinoma [Title/Abstract] OR Gastric cardia adenocarcinoma [Title/Abstract] OR Adenocarcinoma of the cardia [Title/Abstract] OR Adenocarcinoma of the gastric cardia [Title/Abstract] OR Adenocarcinomas of the oesophagogastric junction [Title/Abstract] OR Gastroesophageal junction adenocarcinoma [Title/Abstract] OR Adenocarcinoma of gastroesophageal junction [Title/Abstract] OR Gastric cardia adenocarcinoma [Title/Abstract] OR Esophagogastric junction cancer [Title/Abstract] OR Cardia cancer [Title/Abstract] OR Cancer of the esophagogastric junction [Title/Abstract] OR Esophagogastric junction cancer [Title/Abstract])
**Web of Science**<br>The database was searched on January 1, 2023, n=113.<br>Search Strategy:<br>1 TOPIC: (“Transthoracic” OR “Thoracoabdominal”)<br>2 TOPIC: (“Transabdominal” OR “Transhiatal”)<br>3 TOPIC: (“Esophagogastric junction adenocarcinoma” OR “Adenocarcinoma of the esophagogastric junction” OR “Cardiac adenocarcinoma” OR “Gastric cardia adenocarcinoma” OR “Adenocarcinoma of the cardia” OR “Adenocarcinoma of the gastric cardia” OR “Adenocarcinomas of the oesophagogastric junction” OR “Gastroesophageal junction adenocarcinoma” OR “Adenocarcinoma of gastroesophageal junction” OR “Gastric cardia adenocarcinoma” OR “Esophagogastric junction cancer” OR “Cardia cancer” OR “Cancer of the esophagogastric junction” OR “Esophagogastric junction cancer”)<br>4 #1 AND #2 AND #3 (113)
**EMBASE**<br>The database was searched on January 1, 2023, n=130.<br>Search Strategy:<br>('Transthoracic':ti,ab,kw OR 'Thoracoabdominal':ti,ab,kw) AND ('Transabdominal':ti,ab,kw OR 'Transhiatal':ti,ab,kw) AND ('Esophagogastric junction adenocarcinoma':ti,ab,kw OR 'Adenocarcinoma of the esophagogastric junction':ti,ab,kw OR 'Cardiac adenocarcinoma':ti,ab,kw OR 'Gastric cardia adenocarcinoma':ti,ab,kw OR 'Adenocarcinoma of the cardia':ti,ab,kw OR 'Adenocarcinoma of the gastric cardia':ti,ab,kw OR 'Adenocarcinomas of the oesophagogastric junction':ti,ab,kw OR 'Gastroesophageal junction adenocarcinoma':ti,ab,kw OR 'Adenocarcinoma of gastroesophageal junction':ti,ab,kw OR 'Gastric cardia adenocarcinoma':ti,ab,kw OR 'Esophagogastric junction cancer':ti,ab,kw OR 'Cardia cancer':ti,ab,kw OR 'Cancer of the esophagogastric junction':ti,ab,kw OR ' Esophagogastric junction cancer':ti,ab,kw)
**Cochrane Library**<br>The database was searched on January 1, 2023, n=136.<br>Search Strategy:<br>(“Transthoracic” OR “Thoracoabdominal”): ti,ab,kw AND (“Transabdominal” OR “Transhiatal”): ti,ab,kw AND (“Esophagogastric junction adenocarcinoma” OR “Adenocarcinoma of the esophagogastric junction” OR “Cardiac adenocarcinoma” OR “Gastric cardia adenocarcinoma” OR “Adenocarcinoma of the cardia” OR “Adenocarcinoma of the gastric cardia” OR “Adenocarcinomas of the oesophagogastric junction” OR “Gastroesophageal junction adenocarcinoma” OR “Adenocarcinoma of gastroesophageal junction” OR “Gastric cardia adenocarcinoma” OR “Esophagogastric junction cancer” OR “Cardia cancer” OR “Cancer of the esophagogastric

junction” OR “Esophagogastric junction cancer”): ti,ab,kw - (Word variations have been searched)

Ovid MEDLINE

The database was searched on January 1, 2023, n=1355.

Search Strategy:

1 Transthoracic.ab.
2 Thoracoabdominal.ab.
3 or/1-2 [Transthoracic]
4 Transabdominal.ab.
5 Transhiatal.ab.
6 or/4-5 [Transabdominal]
7 Esophagogastric junction adenocarcinoma.ab.
8 Adenocarcinoma of the esophagogastric junction.ab.
9 Cardiac adenocarcinoma.ab.
10 Gastric cardia adenocarcinoma.ab.
11 Adenocarcinoma of the cardia.ab.
12 Adenocarcinoma of the gastric cardia.ab.
13 Adenocarcinomas of the oesophagogastric junction.ab.
14 Gastroesophageal junction adenocarcinoma.ab.
15 Adenocarcinoma of gastroesophageal junction.ab.
16 Gastric cardia adenocarcinoma.ab.
17 Esophagogastric junction cancer.ab.
18 Cardia cancer.ab.
19 Cancer of the esophagogastric junction.ab.
20 Esophagogastric junction cancer.ab.
21 or/7-20 [Esophagogastric junction adenocarcinoma]
22 3 and 6 and 21 (1355)

ScienceDirect

The database was searched on January 1, 2023, n=69.

Search Strategy:

Title, abstract, keywords: ((“Transthoracic” OR “Thoracoabdominal”) and (“Transabdominal” OR “Transhiatal”) and (“Esophagogastric junction adenocarcinoma” OR “Adenocarcinoma of the esophagogastric junction” OR “Cardiac adenocarcinoma” OR “Gastric cardia adenocarcinoma” OR “Adenocarcinoma of the cardia” OR “Adenocarcinoma of the gastric cardia” OR “Adenocarcinomas of the oesophagogastric junction” OR “Gastroesophageal junction adenocarcinoma” OR “Adenocarcinoma of gastroesophageal junction” OR “Gastric cardia adenocarcinoma” OR “Esophagogastric junction cancer” OR “Cardia cancer” OR “Cancer of the esophagogastric junction” OR “Esophagogastric junction cancer”))

Scopus

The database was searched on January 1, 2023, n=2132.

Search Strategy:

TITLE-ABS-KEY ((“Transthoracic” OR “Thoracoabdominal”) and (“Transabdominal” OR “Transhiatal”) and (“Esophagogastric junction adenocarcinoma” OR “Adenocarcinoma of the esophagogastric junction” OR “Cardiac adenocarcinoma” OR “Gastric cardia adenocarcinoma” OR “Adenocarcinoma of the cardia” OR “Adenocarcinoma of the gastric cardia” OR “Adenocarcinomas of the oesophagogastric junction” OR “Gastroesophageal junction adenocarcinoma” OR “Adenocarcinoma of gastroesophageal junction” OR “Gastric cardia adenocarcinoma” OR “Esophagogastric junction cancer” OR “Cardia cancer” OR “Cancer of the esophagogastric junction” OR “Esophagogastric junction cancer”))

Note: The combined text and medical subject heading (MeSH) terms used were: “Transthoracic”, “Transabdominal” and “Esophagogastric junction adenocarcinoma”.

Table S3 Methodological quality assessments of the included studies.

				Selection					Outcome			
**Study**	**Randomization**	**Masking**	**Accountability of all patients**	**Exposed cohort[a]**	**Nonexposed cohort[b]**	**Ascertainment of exposure**	**Outcome of interest[c]**	**Comparability[d]**	**Assessment of outcome**	**Length of follow-up[e]**	**Adequacy of follow-up**	**Total score**
**Randomized clinical trials**												
2015 Kurokawa [20]	**	**	*									5
2007 Omloo [24]	**	*	*									4
**Cohort studies**												
2022 Oh [10]				*	*	*	*	**	*	*	*	9
2022 Chen [6]				*		*	*	**	*		*	7
2020 Xing [8]				*		*	*	**	*		*	7
2019 Voron [7]				*		*	*	**		*	*	7
2019 Tosolini [13]				*	*		*	**	*		*	7
2019 Reddavid [14]				*	*	*	*	**	*	*	*	9
2018 Yang [11]				*	*	*	*	**	*	*	*	9
2018 Blank [12]				*	*	*	*	**	*		*	8
2016 Zhang [9]				*		*	*	**	*		*	7
2015 Zhou [19]				*		*	*	**	*		*	7
2012 Reeh [21]				*	*	*	*	**	*		*	8
2012 Ovrebo [22]				*	*	*	*	**	*	*	*	9
2010 Zheng [23]				*		*	*	**	*		*	7
1999 Wayman [25]				*	*		*	**	*		*	7
1998 Graham [26]				*	*	*	*	**	*		*	8

Note: [a] Representativeness of the exposed cohort;

[b] Selection of the non-exposed cohort;

[c] Demonstration that outcome of interest was not present at start of study;

[d] Comparability of cohorts on the basis of the design or analysis;

[e] Was follow-up long enough for outcomes to occur.
